# Supplementary material for: Sarracenia alata (Alph.Wood) Alph.Wood Microcuttings as a Source of Volatiles Potentially Responsible for Insects’ Respond
Source: Molecules. 2021 Apr 21;26(9):2406. doi: 10.3390/molecules26092406 (PMC8122457; doi:10.3390/molecules26092406)
Supplement: Supplementary file 1 [file molecules-26-02406-s001.zip › molecules-1158213-supplementary.pdf]

Figures S1-S4 Photographic documentation of the in vivo bioassays

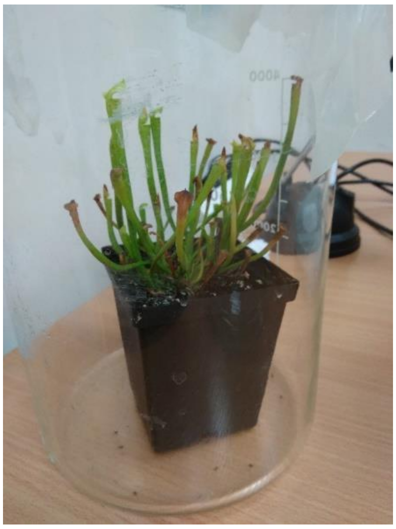

Figure S1 Plant used in the experiment (*Sarracenia alata*) – the glass test vessel after 4 days of exposure (visible dead insects on the bottom)

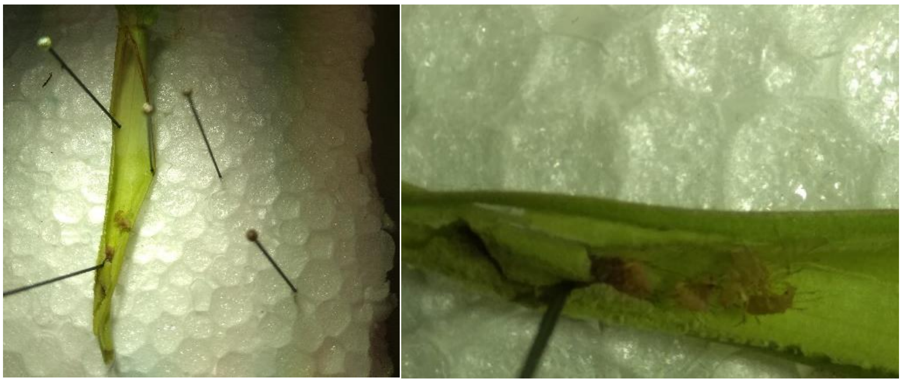

Figure S1 The leaf cut along after two days of exposure with visible aphid bodies

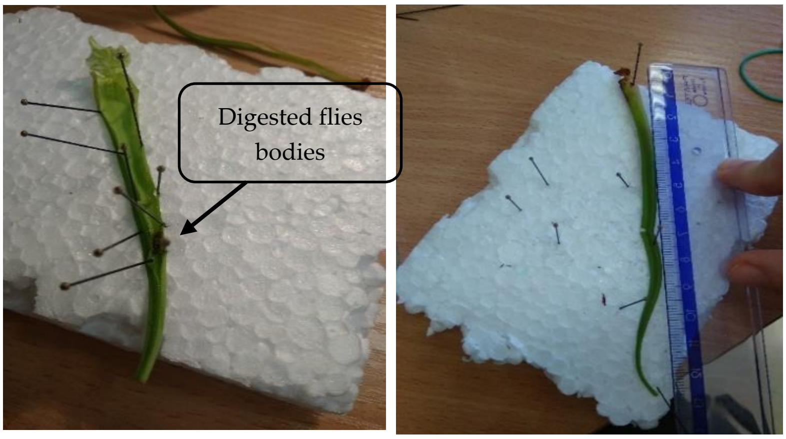

Figure S2 The leaf cut along after four days of exposure with visible digested insects

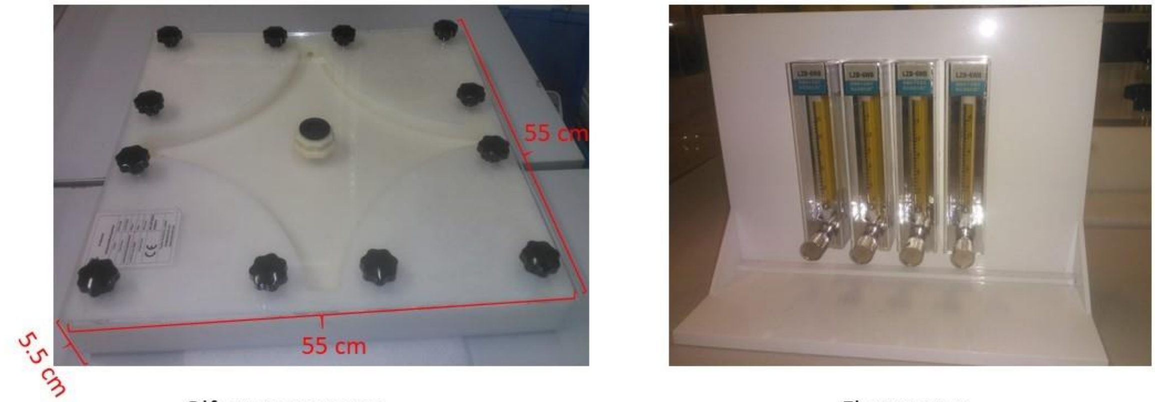

Olfactometer area Flow meter

Figure S4 Olfactometer used in the experiment

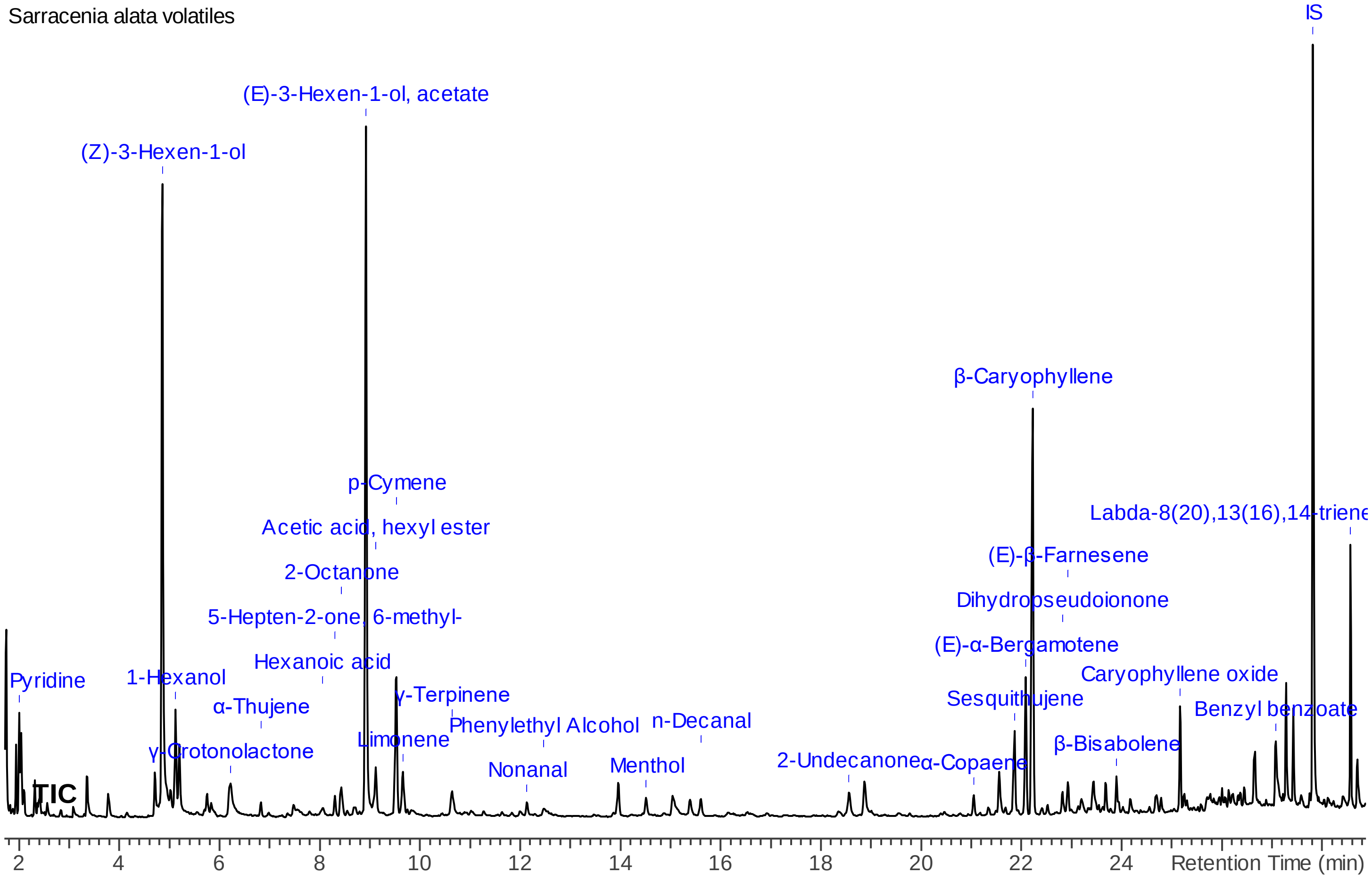

Figure S5 *Sarracenia alata* - volatiles profile (full chromatogram)

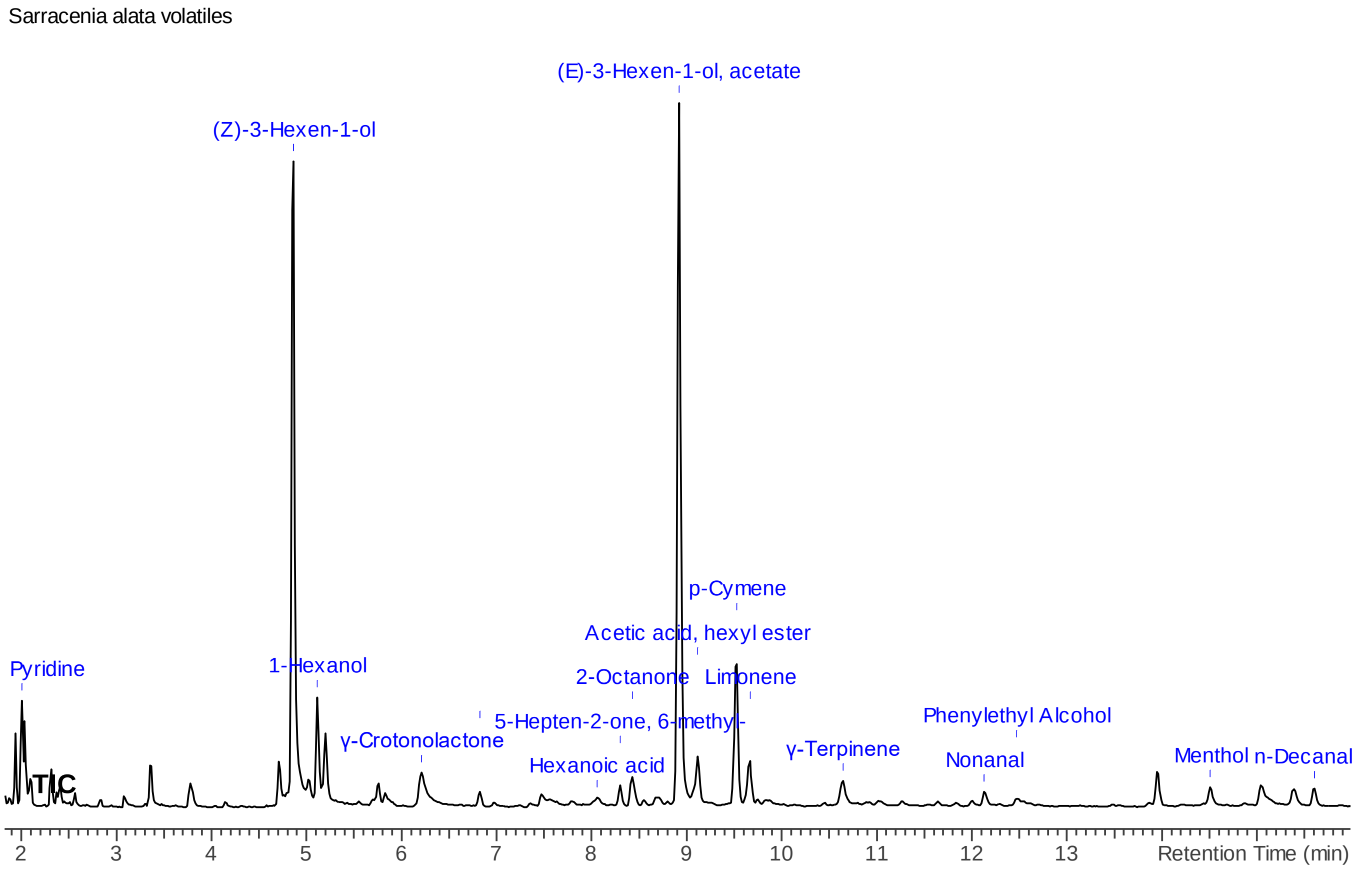

Figure S6 *Sarracenia alata* - volatiles profile (part I: minute 2 - minute 16)

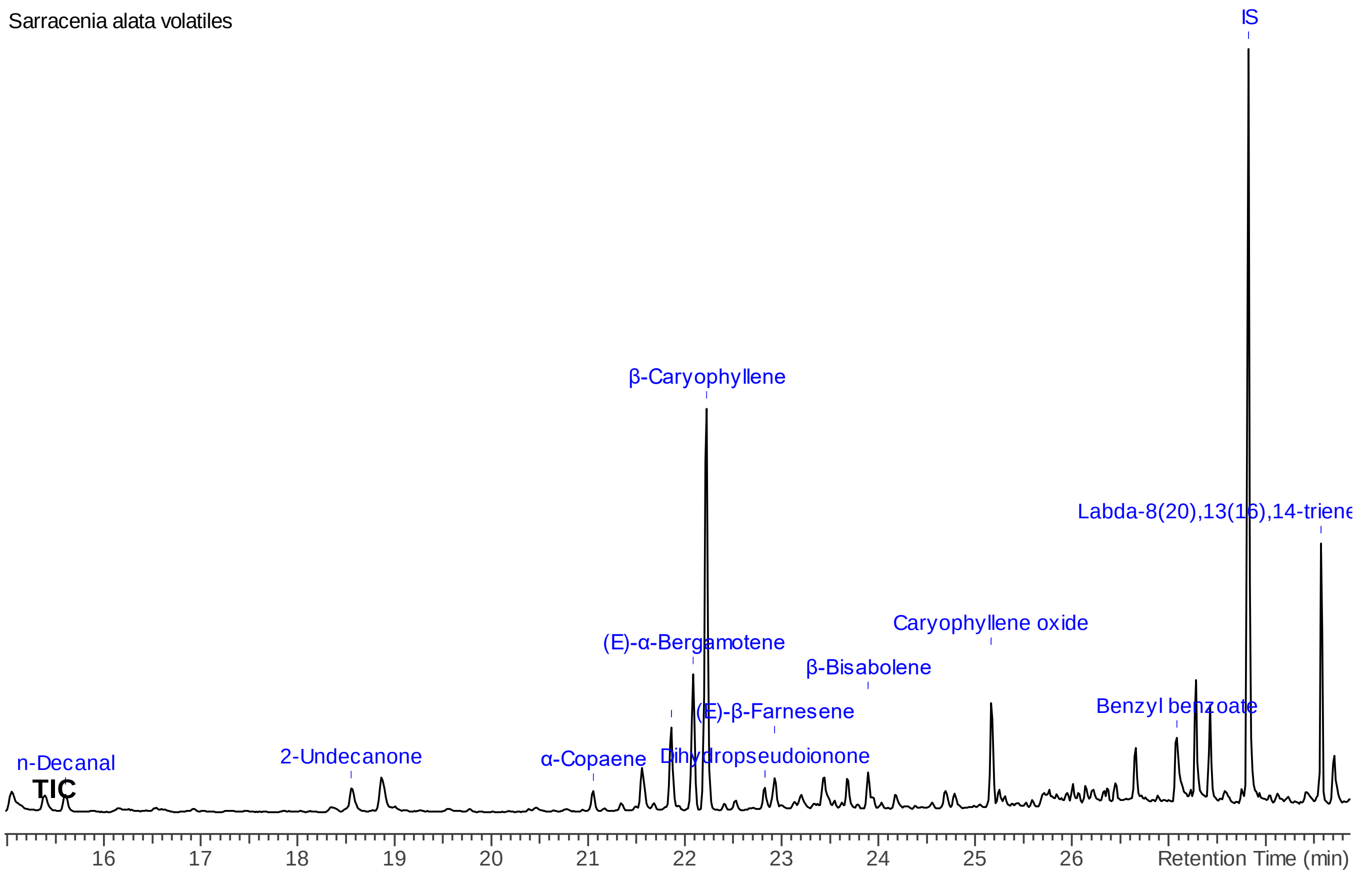

Figure S7 *Sarracenia alata* - volatiles profile (part II: minute 15 - minute 30)

Table S1Particular averages after subsequent passages after twelve weeks I – on media with PGR and on control medium 1/3 MS; II – on media after next fourteen weeks without PGR but earlier explants at thirst step (I) were cultivated on PGR

Passage	Medium	Height of plants		No. of leaves		No. of new shoots		No. of roots		Length of roots		Fresh weight		Dry weight	
I	1/3 MS	5.7±0.24	c	28.6±2.58	ns	5.1±0.43	ns	3.7±0.19	ns	1.26±0.19	ab	0.40±0.01	cdef	0.05±0.00	cdef
I	1/3 MS_0.5BAP + 0.3IAA	5.5±0.47	c	25.3±4.00	ns	5.1±0.76	ns	1.5±0.07	ns	0.58±0.07	d	0.36±0.00	ef	0.05±0.00	def
I	1/3 MS_1BAP + 0.3IAA	6.6±0.25	abc	25.4±1.31	ns	5.1±0.29	ns	2.2±0.08	ns	0.88±0.08	abc	0.56±0.00	abcd	0.07±0.00	abcd
I	1/3 MS_1.5BAP + 0.3IAA	6.3±0.17	abc	29.5±2.96	ns	6.1±0.21	ns	1.9±0.11	ns	1.10±0.11	abc	0.67±0.01	abc	0.09±0.00	ab
I	1/3 MS_2BAP + 0.3IAA	6.2±0.26	abc	30.0±3.35	ns	7.5±0.96	ns	2.3±013	ns	1.12±0.18	abc	0.68±0.05	ab	0.09±0.00	ab
I	1/3 MS_2.5BAP + 0.3IAA	6.8±0.20	abc	27.3±2.03	ns	5.8±0.47	ns	1.9±0.17	ns	0.66±0.15	cd	0.65±0.07	abc	0.08±0.00	ab
I	1/3 MS_3BAP + 0.3IAA	5.8±0.34	bc	26.2±3.44	ns	6.1±0.91	ns	1.5±0.20	ns	0.96±0.08	abc	0.63±0.00	abcd	0.08±0.01	ab
I	1/3 MS_2BAP	6.9±0.27	abc	25.1±2.72	ns	6.7±1.01	ns	1.7±0.19	ns	1.00±0.05	abc	0.70±0.01	ab	0.08±0.00	abcd
I	1/3 MS_0.3IAA	6.4±0.11	abc	24.6±1.03	ns	4.7±0.23	ns	4.6±0.80	ns	1.32±0.14	a	0.53±0.00	abcd	0.06±0.00	abcde
II	1/3 MS	6.4±0.62	abc	17.1±1.79	ns	2.8±0.10	ns	3.5±0.08	ns	1.04±0.04	abc	0.22±0.00	f	0.03±0.00	f
II	1/3 MS_0.5BAP + 0.3IAA	7.6±0.30	a	21.7±1.91	ns	3.9±0.31	ns	1.9±0.26	ns	1.44±0.10	a	0.43±0.00	cdef	0.05±0.00	abcde
II	1/3 MS_1BAP + 0.3IAA	6.7±0.65	abc	20.5±2.24	ns	3.5±0.37	ns	1.8±0.09	ns	1.33±0.11	a	0.57±0.01	abcd	0.08±0.00	abcd
II	1/3 MS_1.5BAP + 0.3IAA	7.1±0.38	abc	24.7±2.25	ns	4.6±0.66	ns	1.6±0.14	ns	1.36±0.12	a	0.62±0.05	abcd	0.08±0.00	abc
II	1/3 MS_2BAP + 0.3IAA	6.1±0.28	abc	23.7±1.39	ns	4.1±0.24	ns	1.9±0.13	ns	0.95±0.11	abc	0.51±0.00	abcd	0.07±0.00	abcd

II	1/3 MS_2.5BAP + 0.3IAA	7.5±0.27	ab	24.7±1.88	ns	4.7±0.56	ns	1.7±0.23	ns	1.24±0.14	ab	0.78±0.04	a	0.10±0.00	a
II	1/3 MS_3BAP + 0.3IAA	6.4±0.17	abc	26.1±1.81	ns	4.7±0.23	ns	1.7±0.25	ns	0.90±0.12	abc	0.70±0.00	ab	0.09±0.00	ab
II	1/3 MS_2BAP	6.6±0.34	abc	22.3±1.12	ns	4.0±0.37	ns	1.8±0.19	ns	0.91±0.11	abc	0.58±0.04	abcd	0.08±0.00	abcd
II	1/3 MS_0.3IAA	5.6±0.30	c	19.6±2.41	ns	3.2±0.34	ns	3.8±0.51	ns	1.23±0.15	ab	0.21±0.02	f	0.03±0.00	ef

significant at p=0.05, abc… Tukey test – two-way of variance analysis. 'ns' interaction media x passages no significant; ±SE – standard error

Table S2 Volatile profile of *Sarracenia alata* microcuttings

**Compound**	**LRI_{exp}**	**LRI_{lit}**	**Contribution [%]**
Pyridine	746	-	3.10±0.07
(*Z*)-3-Hexen-1-ol	864	852	16.48±0.31
1-Hexanol	873	868	2.73±0.12
γ-Crotonolactone	918	914	2.45±0.05
α-Thujene	933	929	0.84±0.11
Hexanoic acid	978	985	1.05±0.09
5-Hepten-2-one, 6-methyl-	985	986	1.05±0.08
2-Octanone	989	990	1.44±0.17
(*E*)-3-Hexen-1-ol acetate	1007	1005	19.99±0.01
Acetic acid, hexyl ester	1011	1012	2.28±0.05
p-Cymene	1026	1025	4.96±0.02
Limonene	1029	1030	2.16±0.12
γ-Terpinene	1058	1060	1.47±0.07
n-Nonanal	1103	1104	0.92±0.06
Phenylethyl Alcohol	1112	1116	1.22±0.10
Menthol	1172	1174	1.16±0.13
Decanal	1206	1204	1.06±0.04
2-Undecanone	1295	1294	1.60±0.10
α-Copaene	1376	1376	1.09±0.19
Sesquithujene	1404	1402	2.91±0.21
(*E*)-α-Bergamotene	1418	1415	4.15±0.19
β-Caryophyllene	1425	1420	11.30±0.27
Dihydropseudoionone	1456	1456	1.02±0.01
(*E*)-β-Farnesene	1458	1458	1.52±0.16
β-Bisabolene	1509	1509	1.57±0.02
Caryophyllene oxide	1590	1581	2.78±0.04
Benzyl Benzoate	1756	1762	2.83±0.18
IS	1850	1858	-
Labda-8(20),13(16),14-triene	1891	1948	4.89±0.09

LRIexp – experimentally obtained linear retention indices; LRI_{lit} – linear retention indices available in literature and databases; Contribution [%] values based on peak area normalization.

Table S3 Insects behaviour during bioassays

**Time**	***Drosophila hydei***	***Acyrthosiphon pisum***
2 hours	-The insects moved chaotically around the test vessel. Few insects sat on the tops of plants, but did not fall into the funnels.	- The aphids were clearly interested in the plant and located mainly on the tips of the leaves,<br>- Some unsuccessful feeding attempts were observed<br>- Individual aphids fell into the funnels.<br>- Leaves from the outside and inside were covered with drops of secretion.
4 hours	-A great part of flies placed on the walls of the vessel. Many were observed on different parts of plants, but not near the funnels.	- Great part of the insects left the plant and placed mainly on the bottom of the glass vessel
2 days	- Few (4) living insects were observed on the walls of the test vessel. No dead insects were found.<br>- After cutting of two leaves, in the largest of them numerous pre-digested flies bodies were found.	- Only few living insects occurred on the plant. Many living aphids on the bottom of the glass vessel.<br>- After cutting two funnels, in the largest leave, about 10 undigested aphids were found.
4 days	-No flies found in the vessel (both living or death).<br>-After cutting of two funnels, in the larger one (about 10 cm), the flesh remains of flies were observed.	- Aphids absent on the plant. Numerous dead individuals at the bottom of the test vessel. After cutting two shoots, in the larger one the digested bodies of several aphids were observed.
Summary	-After 4 days of flies exposure to the *Sarracenia*, almost 100% of the individuals were attracted and digested by the plant. The etched flies were found in the largest leaves (over 10 cm).	During 4 days aphid exposure to the *Sarracenia* plants, it was found that about 50% of the initial number of individuals was attracted to the funnels and digested. Aphids in the initial phase of the test showed changes in behavior (increased activity) and clear interest in the plant. They also made unsuccessful feeding attempts. The remains of aphid bodies were found only in the largest leaves (over 10 cm).
